# Predicting the development of gender-specific premature mortality for Germany by 2030

**DOI:** 10.3205/000289

**Published:** 2021-02-25

**Authors:** Doris Bardehle

**Affiliations:** 1 Stiftung Männergesundheit, Berlin, Germany

**Keywords:** premature mortality, life expectancy, gender equality, men’s health strategy

## Abstract

Germany has set itself the goal of reducing the premature mortality of under 70-year-old men to 190/100,000 and of women to 100/100,000 (age-standardized) by 2030. This is in line with the targets of the United Nations (UN) Sustainable Development Goals (SDG) (2015–2030) to reduce premature mortality by 34% for both men and women during this period.

For the years 2010 to 2018, the premature mortality of 0–69-year-old men and women was calculated and standardized to the European population. On this basis, two linear trend calculations were made and compared with each other: 1. with the data of the target for Germany up to the year 2030, and 2. with the real figures achieved so far.

The goal of reducing premature mortality by 34% within 15 years can, according to the current trend, only be achieved to 13.5% for men and 5.2% for women. Conclusions will be drawn from this as to how premature mortality can be reduced more significantly.

## Zusammenfassung

Deutschland hat sich das Ziel gesetzt, die vorzeitige Sterblichkeit der unter 70-jährigen Männer auf 190/100.000 und der Frauen auf 100/100.000 (altersstandardisiert) bis zum Jahr 2030 zu senken. Das entspricht den Zielstellungen der Nachhaltigen Entwicklungsziele (SDG) der Vereinten Nationen (UN) (2015–2030), die vorzeitige Sterblichkeit in diesem Zeitraum um 34% für Männer und Frauen zu senken.

Für die Jahre 2010 bis 2018 wurde die vorzeitige Sterblichkeit der unter 70-jährigen Männer und Frauen berechnet und auf die Europa-Bevölkerung standardisiert. Auf dieser Grundlage wurden zwei lineare Trendberechnungen vorgenommen und miteinander verglichen: 1. Mit den Daten der Zielstellung für Deutschland bis zum Jahr 2030 und 2. mit den bisher real erreichten Zahlen.

Das Ziel, die vorzeitige Mortalität um 34% innerhalb von 15 Jahren zu senken, kann nach dem gegenwärtigen Trend nur bis 13,5% für Männer und bis 5,2% für Frauen erreicht werden. Daraus werden Schlussfolgerungen abgeleitet, wie die vorzeitige Sterblichkeit stärker abgesenkt werden kann.

## Introduction

As one of 193 countries worldwide [1], Germany committed to the United Nations Sustainable Development Goals, which include a substantial improvement in the health and social situation as well as environmental conditions. The German government has defined the sustainability strategy for Germany on the basis of the UN resolution. The reduction of premature mortality by 34% was in line with the UN Sustainability Strategy and was adopted by Germany for its own sustainability strategy [2]. For Germany, the most important health goal is the reduction of premature mortality for men and women.

The German government has set itself the goal of reducing the premature mortality of under 70-year-old men based on data of 2015 from 288/100,000 to 190/100,000 in the year 2030. For women, the target is to reduce premature mortality from 153/100,000 in 2015 to 100/100,000 in 2030 [2], [3]. The data are age-standardized [3]. This is intended to simultaneously increase the life expectancy of men and women.

The goal of reducing premature mortality by 34% for both men and women within 15 years is critically reviewed. Do we need a strategy for Germany to improve men’s health, the values of which are much worse than those of women? The World Health Organization (WHO) recommends gender-specific strategies [4], [5] such as those already in place in Ireland and Australia. There are efforts by gender medicine, men’s networks and non-governmental organizations (NGOs) in Germany to improve men’s health in a targeted manner.

## Methods

About 20 years ago, premature mortality was defined as age-standardized mortality of men and women under 65 years of age [6] (Annex, p. 296), [7] (p. 737–56), then increased to the population under 70 [6], [8] (p. 20). The current definition [9] includes the population aged 0 to 74 in premature mortality.

The Federal Statistical Office as the publisher of the indicator reports on the sustainable development goals in Germany uses an age structure of 1–69-year-olds with the European standard population of 1976. Special calculations have shown that if the 0–1 age group had been included, the result would have changed by only 1–3/100,000. Thus the calculated figures on premature mortality are internationally comparable.

Premature mortality was calculated by including mortality in the first year of life for 0–69-year-old men and women by 5-year age groups. All calculations were performed using data from the Federal Statistical Office on deceased persons and the mean population [10], [11], including standardization to the European standard population (in use since 1976) itself.

For the standardization, a macro was used, which was worked out within the framework of the indicator set of the federal states [7] (p. 737–56).

The linear trend function was calculated for the years 2010 to 2030 with the Federal government’s goals for the reduction of premature mortality. The linear trend function with the data available since 2010 was calculated taking into account the previous course of premature mortality from 2010 as the starting point of SDG targets and the calculations up to the year 2018.

The linear trend function and its values show the direction and the strength of the annual developments. “x” denotes the decreasing or increasing linear trend per year, and the coefficient of determination characterizes the quality of the correlation of the measured values for a linear trend. The closer the value approaches the number 1, the more meaningful the trend is. The linear trend function and the coefficient of determination were calculated up to the year 2030.

The underlying cause of death was used according to the international death certificate from the Federal Statistical Office. The underlying cause of death is intended to represent the previous illnesses that brought about the immediate cause of death. It is listed on the bottom line in section I of the diseases directly leading to death [12] and coded by the Federal Statistical Office for the unicausal cause of death statistics. No individual diagnoses were used for the evaluation, but rather summarized disease chapters that were coded particularly frequently as underlying causes of death in the context of premature mortality.

## Results

**Figure 1:**
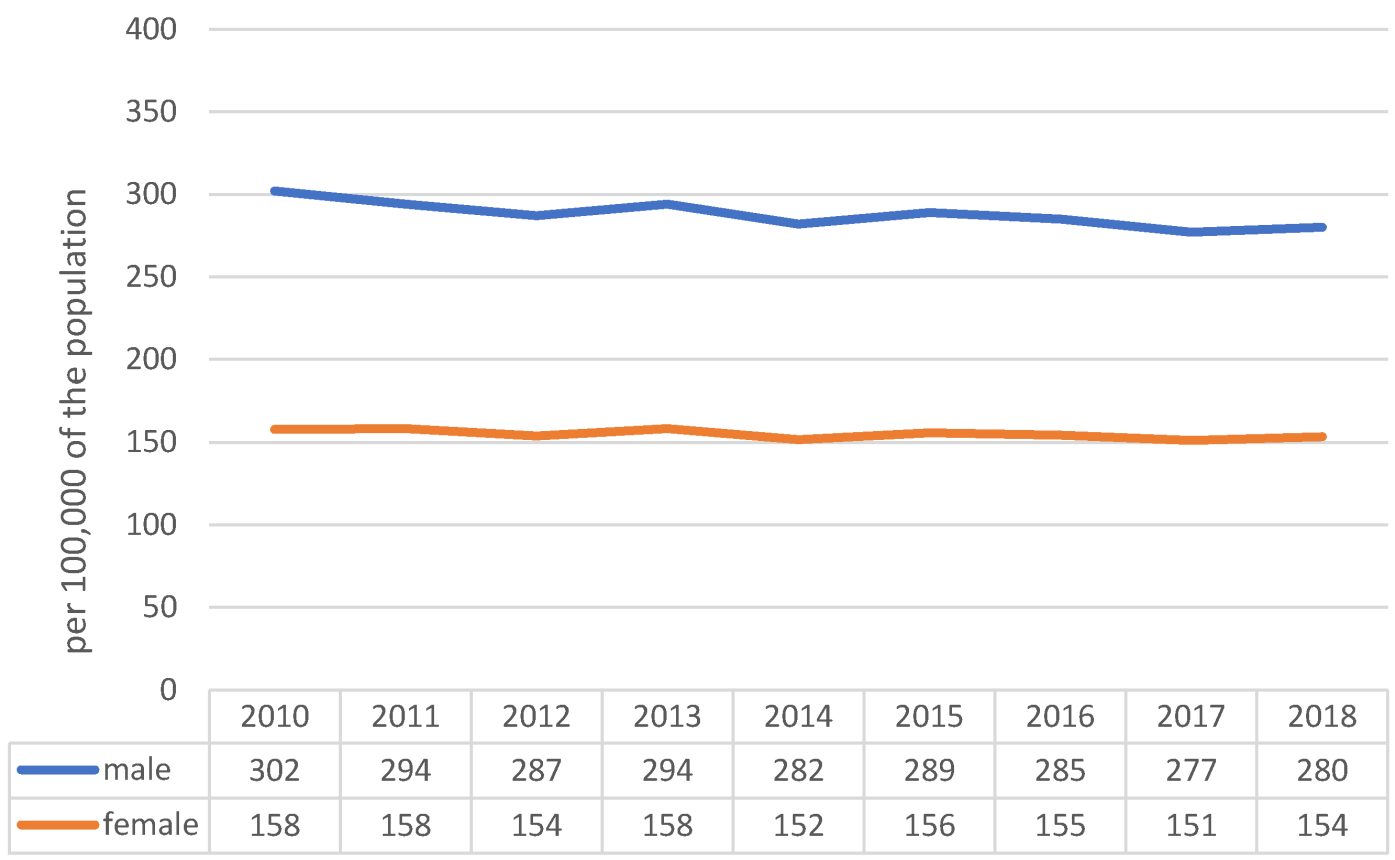
Premature mortality 0–69 years, Germany 2010–2018, standardized to the European population (1976)

From 2010 to 2017, the premature mortality rate for men dropped from 302/100,000 to 277/100,000. In 2018, the rate increased to 280/100,000 (Figure 1 (Fig. 1)). Among women, premature mortality also decreased, from 158/100,000 to 151/100,000, but rose to 154/100,000 in 2018.

### Excess mortality of men over women

A comparison of the gender- and age-specific mortality rates for the year 2018 (Table 1 (Tab. 1)) shows that in each age group, the mortality rates of men are higher than those of women. While in childhood (0–14 years) the percentage of excess mortality among boys is between 5 and 36%, it rises to 72 to 159% in the higher age groups. Men aged 20–29 years are particularly affected by excess mortality. In 2018, a standardized premature mortality rate of 153.50/100,000 for women and 280.40/100,000 for men was achieved, which corresponds to a male excess mortality rate of 82.67% (Table 1 (Tab. 1)).

### Main causes of death of premature mortality

**Figure 2:**
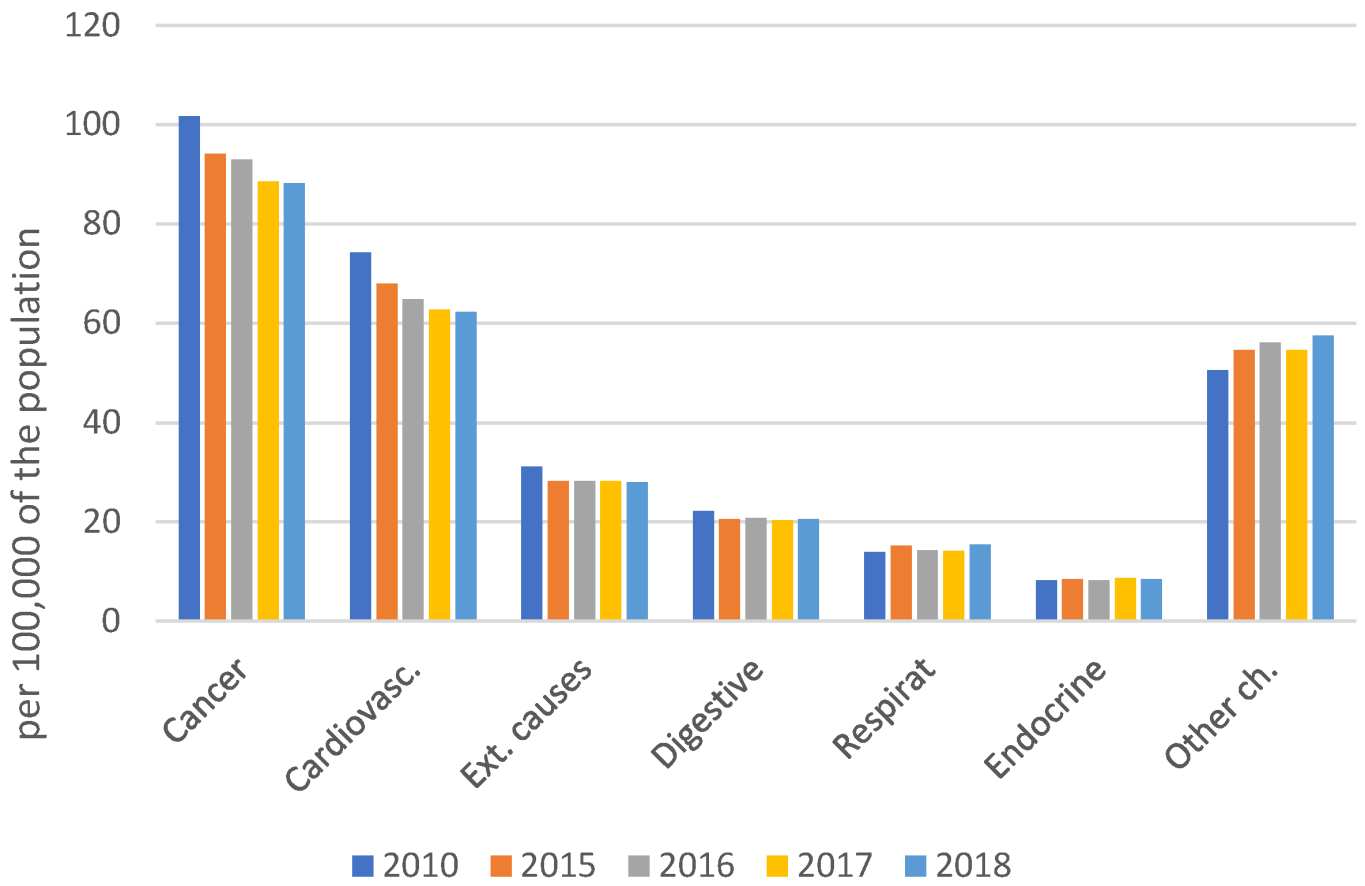
Main causes of premature mortality for men, Germany, 2010 and 2015–2018

Which chapters of diseases (ICD-10) are responsible for the 7% decrease in male mortality within 9 years (Figure 2 (Fig. 2))? Mortality rates have fallen for both men and women due to malignant neoplasms and cardiovascular diseases [10], [11]. Fatal accidents (external causes of mortality) have stagnated for both men and women since 2015. Mortality rates due to diseases of the digestive system and endocrine disorders have stagnated since 2015, too, while mortality rates due to diseases of the respiratory system and other chapters of ICD-10 have increased.

### Trend in premature mortality until 2030

**Figure 3:**
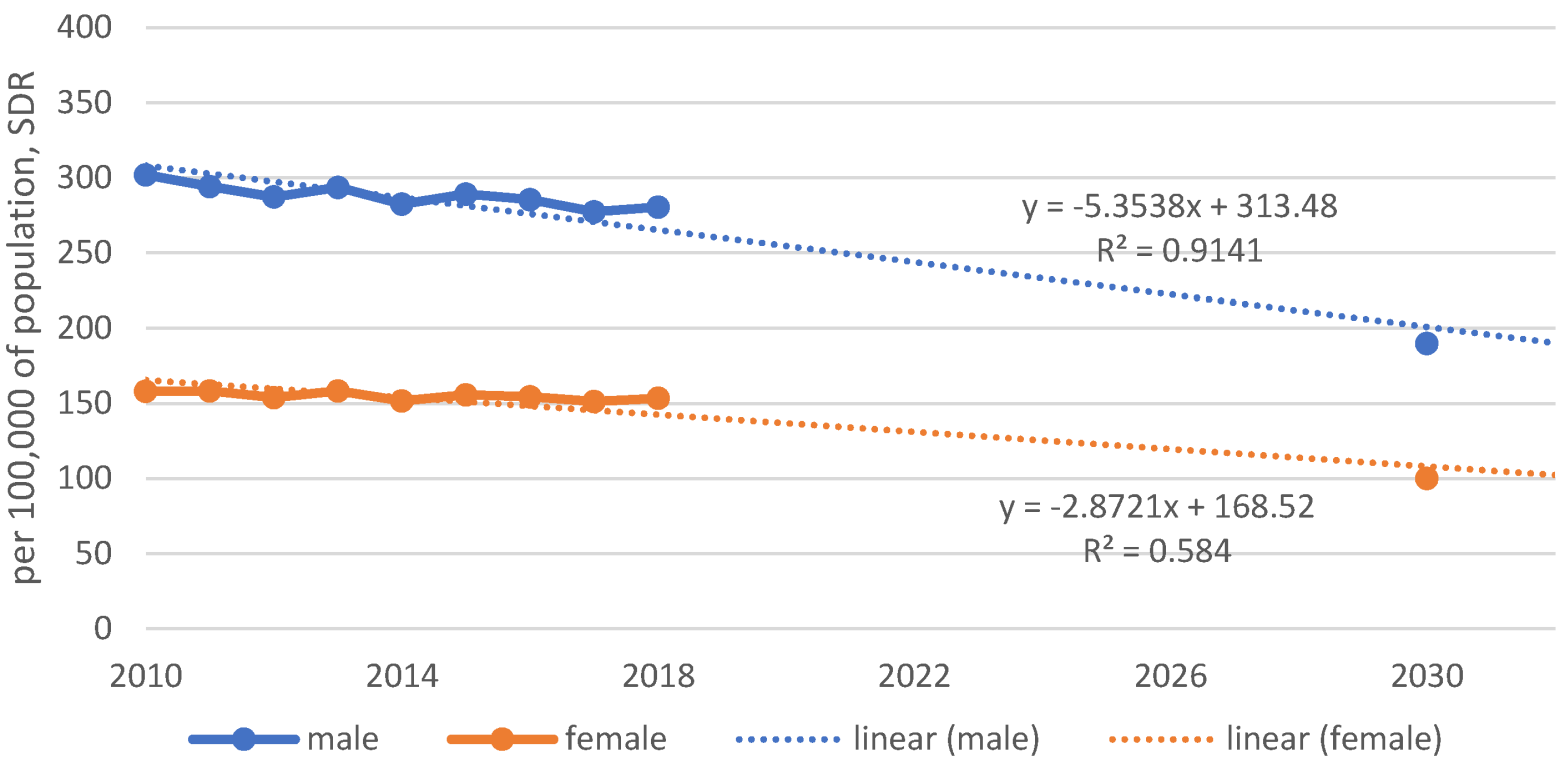
Linear trend function with 34% decrease of premature mortality up to 2030 (190/100,000 for males and 100/100,000 for females)

In order to achieve a 34% reduction in premature mortality by 2030 in line with the SDG targets, a linear trend function was calculated between the baseline value of 2010 and the target value 190/100,000 for men and 100/100,000 for women in 2030 (Figure 3 (Fig. 3)).

The annual target reduction in mortality rates for men was calculated to be 5.35/100,000 and for women 2.87/100,000, thus aiming for a further approximation of mortality rates between men and women. Compared with the target set for Germany, real data from 2010 onwards show a much weaker trend.

**Figure 4:**
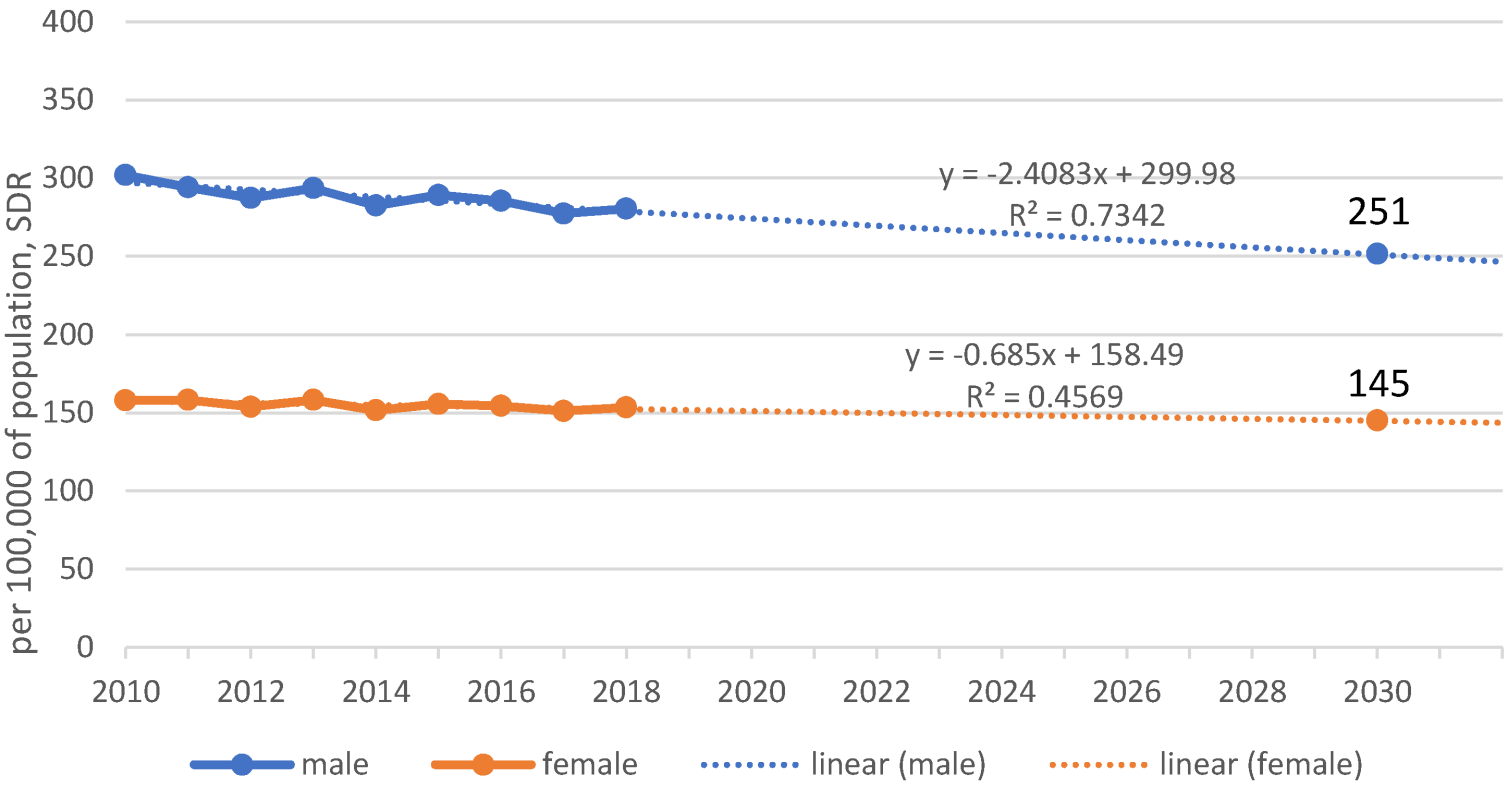
Real situation of reducing premature mortality, Germany, 2010–2030

Currently, we are seeing an average annual decline in mortality rates of 2.4/100,000 for men and 0.7/100,000 for women. A linear trend line derived from these results indicates a possible reduction in mortality rates to 251/100,000 for men and 145/100,000 for women by 2030 (Figure 4 (Fig. 4)).

## Discussion

The Indicator Report 2018 on Sustainable Development in Germany states for indicators 3a and 3b that by 2030, premature mortality should be no more than 100 for women and 190 for men per 100,000 female/male inhabitants [3] (p. 18). There are already indications that, if the trend remains constant, the target for men and women could be missed. Factors cited for a further decline in premature mortality are the decline in chronic diseases, improved health behavior, better medical care and rising health expenditure [3] (p. 18).

It is positive that there has been a decline in premature mortality of 8.7/100,000 among men and 2.3/100,000 among women since the Sustainable Development Goals came into force in 2015. The starting point for the trend calculations is the year 2010. From 2010 to 2018, a decrease in premature mortality of 4.6/100,000 among women was observed, which corresponds to an average annual decrease of 0.51/100,000 deaths among women 0–69 years of age.

For men, premature mortality decreased by 21.5/100,000 in 9 years, corresponding to an annual average reduction of 2.39/100,000 deaths among men aged 0–69. A gender inequality can be seen from the fact that the percentage excess mortality of men was 82.67%, almost twice as high as the mortality of women (Table 1 (Tab. 1)). The differences in age-specific mortality rates have moved only marginally and have not changed the differences in life expectancy of 4.82 years in recent years.

Since 2015, mortality rates for malignant neoplasms and cardiovascular diseases have improved slightly; accidents, digestive and endocrine diseases have stagnated; and diseases of respiratory system and other chapters of ICD-10 have increased (Figure 2 (Fig. 2)). In 2018, a turnaround was observed for the first time, with a small increase in premature mortality for males and females.

A trend function with the current data and its projection shows that instead of 190/100,000 deaths, we will possibly reach around 250/100,000 deaths among men in 2030, and around 145/100,000 deaths among women instead of 100/100,000. This would mean a reduction in premature mortality for men of about 13.5% instead of 34%, and for women it would mean a reduction of only 5.2%.

Due to the COVID-19 pandemic, it can be assumed that there will be no further decrease in premature mortality at least in 2020.

## Conclusions

The analysis of gender-specific premature mortality shows a need for action for Germany to reduce mortality rates and achieve self-imposed goals of the German Sustainability Strategy by 2030.

Without an action plan, equal health opportunities for men and women cannot be achieved [13].

A reduction in premature mortality of up to 40% by 2030 could be realistic if interventions and monitoring according to the analyses of the Assmann Foundation and Norheim et al. supported this process [14], [15].

There should be strategies for the implementation of research results in the practice of health care and its staffing.

To improve prevention services, there could be “men’s health checklists”, which are graded in an understandable form according to age groups and can be checked and documented by general practitioners, specialists, urologists, psychologists, and company doctors in the form of teamwork [16].

## Notes

### Competing interests

The author declares that she has no competing interests.

## Figures and Tables

**Table 1:**
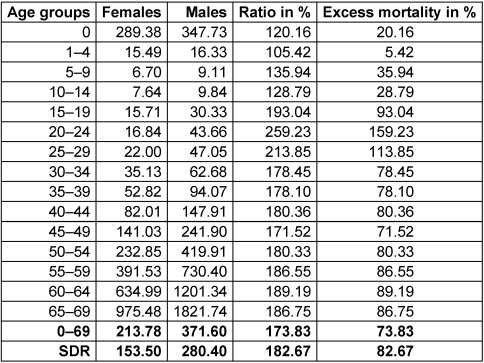
Age and gender distribution of premature mortality in Germany, 2018, excess mortality of men [17]; own calculations
